# Animal Models for Coffin-Lowry Syndrome: RSK2 and Nervous System Dysfunction

**DOI:** 10.3389/fnbeh.2018.00106

**Published:** 2018-05-23

**Authors:** Matthias Fischer, Thomas Raabe

**Affiliations:** ^1^Department of Psychiatry, Psychosomatics and Psychotherapy, Center of Mental Health, University Hospital of Würzburg, Würzburg, Germany; ^2^Institute of Medical Radiation and Cell Research, Biozentrum, University of Würzburg, Würzburg, Germany

**Keywords:** Coffin-Lowry syndrome, Rsk2, mental disorders, mouse model, *Drosophila* model, neuronal dysfunction, behavior

## Abstract

Loss of function mutations in the *rsk2* gene cause Coffin-Lowry syndrome (CLS), which is associated with multiple symptoms including severe mental disabilities. Despite the characterization of ribosomal S6 kinase 2 (RSK2) as a protein kinase acting as a downstream effector of the well characterized ERK MAP-kinase signaling pathway, it turns out to be a challenging task to link RSK2 to specific neuronal processes dysregulated in case of mutation. Animal models such as mouse and *Drosophila* combine advanced genetic manipulation tools with *in vivo* imaging techniques, high-resolution connectome analysis and a variety of behavioral assays, thereby allowing for an in-depth analysis for gene functions in the nervous system. Although modeling mental disability in animal systems has limitations because of the complexity of phenotypes, the influence of genetic variation and species-specific characteristics at the neural circuit and behavioral level, some common aspects of RSK2 function in the nervous system have emerged, which will be presented. Only with this knowledge our understanding of the pathophysiology of CLS can be improved, which might open the door for development of potential intervention strategies.

## Introduction

Coffin-Lowry syndrome (CLS, OMIM 303600) is a rare X-chromosome linked disorder with an incidence of 1:50,000–100,000. Clinical characteristics are heterogeneous and variable in expressivity. They include facial dysmorphism, digit and skeletal abnormalities, and growth delay. Prominently, CLS patients suffer from severe mental disabilities (IQ: 15–60). Less frequently, stimulus-induced drop attacks, epileptic seizures and hearing loss are manifested. The risk to develop psychiatric diseases like depression and psychosis might be increased. No treatment exists for this disease (Pereira et al., 2010). CLS is caused by loss-of-function mutations in the p90 ribosomal S6 kinase 2 (RSK2), which acts as one of many downstream effectors of the MAP-kinase ERK in the RAS-RAF-MEK-ERK signaling pathway. Four RSK isoforms (RSK1–4) are expressed in vertebrates. The identification of multiple phosphorylation substrates implicated RSK proteins as important regulators of transcription, chromatin organization, translation, cell proliferation, migration and survival (Romeo et al., 2012; Cho, 2017). This generalized view raises questions about redundancy and isoform-specific targets in the nervous system, involvement of RSK2 in cellular and neurophysiological processes, and how RSK2 loss-of-function causes distinct neuronal deficits in CLS patients. To address these points, *RSK2* knock-out mice (*RSK2*^−^) and mutants of the single *RSK* ortholog in *Drosophila* (*D-RSK*) were analyzed at the behavioral and neurophysiological level. We summarize findings with both animal models and their implications to better understand the neuropathophysiology of CLS.

## RSK Proteins: Structural Organization, Regulation and Effects on ERK Signaling

**Figure 1:**
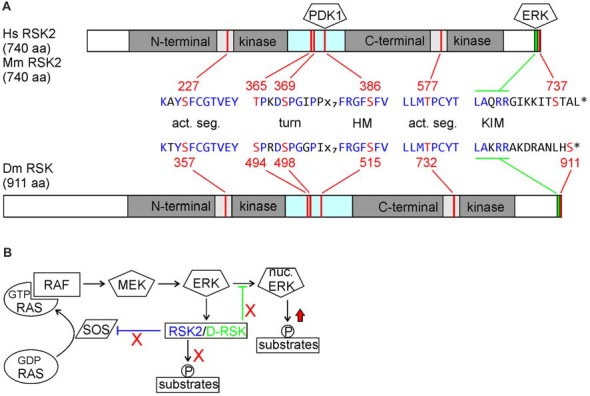
**(A)** Comparison of human and mouse ribosomal S6 kinase 2 (RSK2) with *Drosophila* D-RSK. Conservation of all relevant phosphorylation sites (red) embedded in common consensus sequences (blue) indicates a common mode of activation. Activated ERK binds to the C-terminal kinase interaction motif (KIM) and induces catalytic activity of the C-terminal kinase domain (CTKD) by phosphorylation of a threonine residue (RSK2: T577; D-RSK: T732) in the kinase activation segment. The CTKD in turn phosphorylates a serine residue (RSK2: S386; D-RSK: S515) in the hydrophobic motif (HM) located in the linker region, which promotes binding and activation of 3-phosphoinositde-dependent kinase 1 (PDK1). Furthermore, ERK phosphorylates two residues (RSK2: T365, S369; D-RSK: S494, S498) in the turn motif next to the N-terminal kinase domain (NTKD). In combination with PDK1-mediated phosphorylation of serine S227 (D-RSK: S357) this stabilizes the active conformation of the NTKD, as shown for other AGC-type kinases (Leroux et al., 2018). Involvement of *Drosophila* PDK1 in D-RSK activation was deduced from genetic interaction studies (Rintelen et al., 2001). Release of ERK is promoted by NTKD-mediated autophosphorylation of serine 737. Whether the corresponding C-terminal serine residue 911 in D-RSK has a similar function is not known. **(B)** Integration of RSK2 and D-RSK in MAP-kinase signaling. Loss-of-function mutations not only abolish phosphorylation of RSK substrate proteins but also prevent feedback inhibition (red crosses) resulting in enhanced ERK-mediated phosphorylation of substrate proteins (red arrow). Different mechanisms for negative regulation of ERK by RSK2 (inhibition of RAS activation) or D-RSK (inhibition of ERK nuclear translocation) have been described.

RSK proteins are characterized by two kinase domains separated by a linker region: the N-terminal kinase domain (NTKD) belongs to the AGC kinase family and the C-terminal kinase domain (CTKD) is related to the Ca^2+^/calmodulin-dependent protein kinase (CAMK) family. With the exception of a 140 amino acid N-terminal extension, D-RSK shows equal sequence similarities of about 70% to RSK1–4. Notably, all sequence motifs required for RSK activation are also present in D-RSK (Figure 1A). From biochemical studies a sequential activation mechanism of RSK proteins was deduced, where binding of ERK stimulates CTKD activity, which in turn is necessary for NTKD activation as the effector kinase for phosphorylation of substrate proteins (Romeo et al., 2012; Lara et al., 2013). However, one report in *Drosophila* provided evidence that D-RSK can act independent of catalytic activity of the NTKD in the circadian clock (Tangredi et al., 2012).

RSK proteins not only act as downstream effectors of ERK but reversely influence also ERK activity or localization. During *Drosophila* eye development, D-RSK acts as a cytoplasmic anchor for ERK, thereby inhibiting ERK nuclear translocation and phosphorylation of nuclear targets (Kim et al., 2006). For vertebrate RSK2, negative feedback regulation of ERK activity involves phosphorylation and thereby inactivation of the RAS guanine nucleotide exchange factor SOS and stimulation of the GTPase activating protein (GAP) NF1 (Douville and Downward, 1997; Saha et al., 2012; Hennig et al., 2016). In the nervous system, up-regulation of ERK activity has been verified in the hippocampus and motoneurons of *RSK2*^−^ mice (Fischer et al., 2009a; Schneider et al., 2011) as well as in larval motoneurons and adult brains of *D-RSK* mutants (Fischer et al., 2009b; Beck et al., 2015). Thus, besides acting as a downstream effector of ERK, negative regulation of ERK signaling is an apparent common feature of RSK2 and D-RSK. Yet, the mild increase of ERK activity observed in *RSK2* or *D-RSK* knock-out animals suggests a more modulatory effect of RSK proteins on ERK signaling. In support of a negative regulatory function of RSK2 on ERK also in humans, a patient originally diagnosed at childhood with Noonan syndrome (the most common form of RASopathies, resulting in mild hyperactivation of RAS/ERK signaling) carried a mutation in *RSK2* and later on developed clearer characteristics of CLS, which generally resemble those of RASopathies (short stature, facial dysmorphia, cardiac defects and developmental delay; Chen et al., 2014).

The dual role of RSK2/D-RSK as downstream effectors and negative regulators of MAP-kinase signaling has important implications to explain neuronal dysfunction. Phenotypes could arise because of failure to mediate ERK signals to downstream targets. On the other hand, missing negative regulation of ERK activity might result in elevated or sustained phosphorylation of other ERK target proteins (Figure 1B). The opposing functions of RSK might be of different valence in a certain cellular context. Therefore, one important and challenging point for understanding the pathophysiology of CLS is to distinguish between loss- and/or gain-of function effects on local ERK-mediated signaling.

## Loss of RSK2 and D-RSK Affects Various Cognitive and Emotional Behaviors

The similarities of molecular pathways controlling the generation and differentiation of neurons, neural circuit wiring and synaptic communication make animal models a powerful tool to characterize cellular and physiological processes and link them to human disorders. A more challenging task is to model mental disabilities or mental health disorders in animal systems because of the complexity of symptoms, the influence of genetic variation on disease outcome and species-specific characteristics at the molecular, neural circuit and behavioral level (Androschuk et al., 2015; Leung and Jia, 2016). Nevertheless, a variety of spatial and associative learning assays were established in mice and *Drosophila* to test for cognitive functions. “Emotional” behavior like trait anxiety can be tested in mice with elevated plus maze, open field and light/dark box, whereas locomotion of flies close or at distance to a surrounding wall is considered as anxiety-like behavior. In both animal systems, unavoidable punishment (learned helplessness) induces features of depression, expressed as general inactivity. Furthermore, activity recordings allow correlating disease associated genes with circadian and sleep disturbances as one characteristic of some mental illnesses.

Several hypomorphic, partial deletion and complete loss-of-function* D-RSK* alleles were isolated in a screen for mutations affecting operant space learning and associative olfactory learning in flies (Putz et al., 2004). Complexity arises by the finding that operant learning was not affected in complete knock-out animals but was impaired in a dominant manner by up- or downregulation of *D-RSK* gene dosage or by deletion of the NTKD. Impairment of associative olfactory learning is a recessive trait and expressivity correlates with reduced or complete absence of D-RSK (Putz et al., 2004). It remains to be verified whether D-RSK is specifically required in the mushroom body structure in the fly brain as the center for olfactory memory formation, and if so, to determine its role in the different steps of information processing. D-RSK also plays a role in visual orientation memory and this function was mapped to the ellipsoid body of the central complex of the adult brain (Neuser et al., 2008). An involvement of D-RSK for other behaviors controlled by the central complex, including visual place learning and pattern discrimination, needs to be verified. *D-RSK* mutants also show changes in circadian activity rhythms; instead of maintaining a 24-h rhythm in constant darkness, they display a 23-h periodicity (Akten et al., 2009). This function of D-RSK was mapped to master clock neurons in the brain and involves phosphorylation of the GSK3β homolog Shaggy as a key regulator of the *Drosophila* molecular clock (Beck et al., 2018).

Two different *RSK2*^−^ mouse lines were used for behavioral analyses. Coordination problems (Dufresne et al., 2001), spatial learning as well as long-term memory and spatial working memory (Dufresne et al., 2001; Poirier et al., 2007) and fear conditioning defects were observed (Morice et al., 2013). Differences in circadian behaviors were not detected in long-term testing in the IntelliCage (Fischer et al., 2017). Many cognitive defects were modest, and learning was normal in other paradigms, including spatial learning in the IntelliCage, possibly because there is no need to adapt to new test environments like in conventional paradigms (Fischer et al., 2017). So, compared to often severely affected male CLS patients, cognition in male hemizygous *RSK2*^−^ mice is only mildly impaired in specific situations. If other RSK isoforms can compensate for loss of RSK2 in this mouse model is unknown. *RSK1–3* triple-knock-out mice (Dumont et al., 2005; Laugel-Haushalter et al., 2014) have not been tested in behavior so far. The focus of research was not only on cognitive functions, however. Emotional behavioral phenotypes have also been investigated and included signs of increased spontaneous activity without changes in impulsivity (Poirier et al., 2007; Fischer et al., 2017), an anti-depressive, sucrose reward seeking phenotype, reduced anxiety (Fischer et al., 2017) and changes in value-based contextual conditioning (Darcq et al., 2011). Although some of these phenotypes were not always consistently observed with the different experimental set-ups in the various studies, they correlate with pronounced expression of mouse *RSK2* *mRNA* in learning-relevant regions. These include CA1–3 of the hippocampus, layers V and VI of the neocortex, Purkinje cells and cells of the pyriform cortex (which provide a link to the amygdala) and the habenula, playing a role in motivational and rewarding aspects of behavior (Darcq et al., 2011). A recent study uncovered deficits in spatial pattern separation in adult *RSK2*^−^ mice (Castillon et al., 2018). There is evidence from other studies that this hippocampus-dependent form of memory is promoted by integration of dentate gyrus derived adult-born neurons into existing neural circuits (Goncalves et al., 2016; Anacker and Hen, 2017).

In adult human brain, RSK2 is expressed in the cerebellum, the occipital pole and the frontal lobe (Zeniou et al., 2002). In human embryonic tissue it is also expressed in the hippocampus anlagen (Guimiot et al., 2004). Memory defects in CLS patients have been reported like in the animal models, being more severe in hemizygous males than in heterozygous females (Simensen et al., 2002). However, if the observed changes in emotional behavior in mice are also paralleled in CLS patients is not known so far due to lack of knowledge about psychopathological phenotypes. Based on personal observations (M. Fischer), there is no pronounced hyperactive, anxious or depressive phenotype in these patients.

## RSK2 and D-RSK Influence Pleiotropic Neuronal Properties

**Figure 2:**
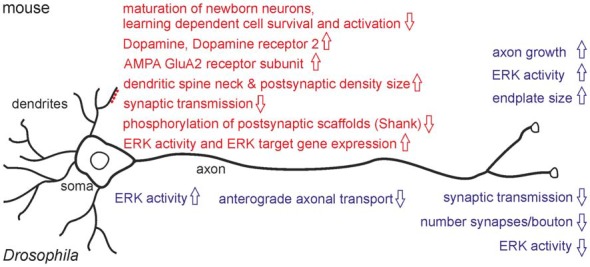
Major neuronal phenotypes observed in *RSK2* knock-out mice (upper part) and *D-RSK* mutants (lower part). Motoneuron phenotypes are indicated in blue, effects on CNS neurons with their dendritic spines are highlighted in red.

Do the behavioral phenotypes seen in mice and *Drosophila* relate to neuronal dysfunction? While *RSK2/D-RSK* knock-outs showed no gross anatomical brain abnormalities in adults, magnetic resonance imaging (MRI) from CLS patients uncovered a reduction in total brain volume with a particular impact on temporal lobe, cerebellar and hippocampal volumes (Kesler et al., 2007). Whether reduced prenatal neurogenesis or neuron loss is a reason for the brain volume loss in humans is unknown. Changes in neurogenesis are evident during mouse embryogenesis, where a reduction in differentiation of cortical radial glia progenitor cells to neurons was observed (Dugani et al., 2010). A recent study verified alterations in adult neurogenesis in the dentate gyrus of the hippocampus of *RSK2*^−^ mice and associated these changes with memory deficits (Castillon et al., 2018). Adult neural stem cells residing in the subgranular zone of the dentate gyrus produce intermediate progenitors and neuroblasts, which finally differentiate into dentate gyrus granule neurons. Because hippocampal neurogenesis is stimulated in an experience-dependent manner and newly generated neurons have distinct characteristics, their integration at a critical maturation stage into hippocampal neural circuits provides plasticity in response to environmental changes (Goncalves et al., 2016; Anacker and Hen, 2017). Under naïve conditions, *RSK2*^−^ mice showed delayed maturation of newly generated neurons with no influence on the final number of mature newborn neurons. Spatial learning had a negative influence on the number of proliferating cells and did not increase the number of newborn neurons like in wild-type. In comparison to wild-type, spatial memory recall in *RSK2*^−^ mice activated less newborn neurons (immediate early gene Zif268 positive) but more pre-existing neurons (Castillon et al., 2018; Figure 2). These experiments provided first evidence that altered adult neurogenesis can contribute to the behavioral phenotypes seen in *RSK2*^−^ mice in a context-dependent manner.

Loss of RSK2 interferes at several levels with neuronal properties (Figure 2). Expression of genes important for synaptic plasticity as a basis for learning is altered in the hippocampus of *RSK2*^−^ mice (Mehmood et al., 2011; Morice et al., 2013). Defects in neurite outgrowth, neuronal and synaptic plasticity could be a major reason for neurocognitive dysfunctions as well. Cultured cortical neurons from *RSK2*^−^ animals showed a growth and developmental delay (Ammar et al., 2013). Dysregulated neurite growth was demonstrated in isolated motoneurons, but survival was unaffected. Motoneuron endplate size was increased *in vivo* (Fischer et al., 2009a). Importantly, sub-structural neuronal alterations were also found in the dentate gyrus. Morphology, but not density, of excitatory synapses is affected as seen by an increase in dendritic spine neck size and enlargement of postsynaptic densities (Morice et al., 2013). These changes might reflect a compensatory mechanism for reduced neurotransmission since electrophysiological recordings for LTP are normal (Morice et al., 2013). On the other hand, altered spine morphology has been associated with several cognitive disorders (Volk et al., 2015), thus it might relate more directly to *RSK2*^−^ behavioral deficits.

Accumulating data support the view that RSK2 influences ionotropic glutamatergic synapses. A focus of RSK2 research is the AMPA-subfamily of glutamate receptors because of their fundamental role in synaptic plasticity as a crucial step for learning and memory formation. The number of AMPA-receptors and their channel properties are influenced by *de novo* synthesis, trafficking, variations in heterotetrameric subunit composition, posttranslational modifications and interaction with scaffold proteins at postsynaptic densities (Huganir and Nicoll, 2013; Chater and Goda, 2014). In the hippocampus of *RSK2*^−^ mice, upregulated levels of the GluA2 subunit of AMPA-receptors at synaptic sites were observed (Mehmood et al., 2011). Increased GluA2 gene expression correlated with elevated ERK-mediated transcriptional activity explained by missing RSK2 feedback inhibition (Schneider et al., 2011; Mehmood et al., 2013). Changes in AMPA receptor subunit composition might affect channel properties or activity dependent synaptic changes. Electrophysiological recordings in *RSK2*^−^ mice or from hippocampal slices demonstrated decreased synaptic transmission, altered AMPA and NMDA receptor properties, but normal paired-pulse facilitation and long-term potentiation. Altogether, these findings argued for a postsynaptic function of RSK2 in neurotransmission (Morice et al., 2013). Distinct phenotypes were also observed in cortical neurons by expression of a dominant-negative acting kinase-deficient variant of RSK2 (Thomas et al., 2005). The frequency of AMPA receptor mediated miniature excitatory postsynaptic currents (mEPSC) was reduced. Although this phenotype could be explained by decreased neurotransmitter release from presynaptic terminals, an alternative model suggested alterations in the number or organization of synaptic contacts (Thomas et al., 2005).

Another function of RSK2 at the postsynapse is interaction with proteins of the Shank family, which act as major scaffolds for AMPA-, NMDR- and metabotropic glutamate receptors (Sala et al., 2015). RSK2 binds and phosphorylates Shank 1 and Shank 3 in response to NMDR-receptor induced activation of ERK signaling (Thomas et al., 2005). RSK2 dependent elevated phosphorylation levels of Shank proteins were also observed in a mouse model for Fragile X-syndrome (FXS). In these mice, a link between increased ERK/RSK activity in the neocortex and audiogenic seizure susceptibility was recognized (Sawicka et al., 2016). A further link between RSK2 and Shank was established by Morice et al. (2013). LTP-induced elevated expression of Shank3 was abolished in *RSK2*^−^ mice. Thus, RSK2 seems to mediate at least some of its effects by modulation of scaffold proteins of the postsynaptic density in an activity-dependent manner.

Emotional behaviors like anxiety, depression or hyperactivity are regulated by the neurotransmitters dopamine and serotonin. Dopamine levels in the mouse cortex of *RSK2*^−^ animals are increased with an accompanying up-regulation of dopamine receptor2 expression (Pereira et al., 2008), while in brain subregion-specific analyses only changes of dopamine metabolites were detected (Fischer et al., 2017). RSK2 also interacts with the serotonergic system by phosphorylating the 5-HT_2A_ receptor, thereby linking growth factor induced MAP-kinase signaling with regulation of a G-protein coupled receptor (Sheffler et al., 2006; Strachan et al., 2009, 2010). For serotonin and its metabolite, only non-significant trends in *RSK2*^−^ brains were detected (Pereira et al., 2008; Fischer et al., 2017).

In *Drosophila*, analysis has focused on the larval neuromuscular system as a well-established model for synapse formation, neurotransmission and synaptic plasticity (Harris and Littleton, 2015). Motoneurons form endings called boutons, each harboring a number of synaptic sites with presynaptic active zones and opposed postsynaptic densities containing ionotropic glutamate receptors (GluRs). In *D-RSK* mutants, overall bouton number is increased but this phenotype is counteracted by a much stronger decrease in synapse number per bouton (Fischer et al., 2009b; Beck et al., 2015). Whether the synaptic phenotypes correlate with the observed anterograde axonal transport defects in motoneurons must be verified. Interestingly, loss of D-RSK resulted in reduction of activated ERK in motoneuron axons and synaptic sites, but in elevated ERK activity in the somata (Beck et al., 2015). Thus, even in a single cell, loss of D-RSK can have opposing effects on ERK signaling in different subcellular compartments. At the physiological level, impaired synaptic transmission was recorded. Notably, the postsynaptic response to a single synaptic vesicle release event was reduced, indicating a postsynaptic function of D-RSK (Beck et al., 2015). Thus, an emerging common feature of mouse RSK2 and *Drosophila* D-RSK is a postsynaptic requirement for efficient synaptic transmission.

## Translational Implications

While no information exists about the neuronal basis of the clinical phenotype in CLS patients, recent findings in mouse and *Drosophila* gave first insights into the impact of RSK2 on neuronal functions. In CLS patients it is still unknown, if similar neurite growth and synaptic defects exist like in animal models. Patient derived iPSCs could be differentiated into distinct neuron subtypes and analyzed at the molecular, biochemical and physiological level. Besides analysis of molecular interactions, a particular emphasis for further studies should be the analysis of RSK2 in experience-dependent synaptic changes. A good example for future research about CLS is the fragile-X mental retardation syndrome, where parallel experiments in animal models have paved the way to bridge the gap to better understand the human disease phenotypes. For example, metabotropic glutamate receptor and ERK-pathway dependent protein synthesis were affected, which led to clinical trials in patients with a mGluR5 antagonist, unfortunately turning out to be not effective (Berry-Kravis et al., 2018). Animal models as platforms for drug screenings might provide an entry point for therapeutic approaches also in CLS. Given the complexity of *RSK2/D-RSK* phenotypes, our understanding of neuronal dysfunction is far from complete at all levels of analysis.

## Author Contributions

MF and TR wrote the manuscript.

## Conflict of Interest Statement

The authors declare that the research was conducted in the absence of any commercial or financial relationships that could be construed as a potential conflict of interest.
